# Supplementary material for: Enhancing Mechanical and Antimicrobial Properties of Dialdehyde Cellulose–Silver Nanoparticle Composites through Ammoniated Nanocellulose Modification
Source: Molecules. 2024 Apr 30;29(9):2065. doi: 10.3390/molecules29092065 (PMC11085600; doi:10.3390/molecules29092065)
Supplement: Supplementary file 1 [file molecules-29-02065-s001.zip › molecules-2962012-supplementary.pdf]

Supporting Information

Enhancing Mechanical and Antimicrobial Properties of Dialdehyde Cellulose–Silver Nanoparticle Composites through Ammoniated Nanocellulose Modification

Jinsong Zeng [1,2], Chen Wu [1,2], Pengfei Li [1,2,3,*], Jinpeng Li [1,2], Bin Wang [1,2], Jun Xu [1,2], Wenhua Gao [1,2] and Kefu Chen [1,2]

[1] Plant Fibril Material Science Research Center, State Key Laboratory of Pulp and Paper Engineering, School of Light Industry and Engineering, South China University of Technology, Guangzhou 510640, China; fezengjs@scut.edu.cn (J.Z.); 202220127983@mail.scut.edu.cn (C.W.); ljp@scut.edu.cn (J.L.); febwang@scut.edu.cn (B.W.); xujun@scut.edu.cn (J.X.); segaowenhua@scut.edu.cn (W.G.); ppchenkf@scut.edu.cn (K.C.)

[2] Guangdong Provincial Key Laboratory of Plant Resources Biorefinery, Guangzhou 510006, China

[3] School of Environment and Energy, South China University of Technology, Guangzhou 510640, China

* Correspondence: felpf@scut.edu.cn; Tel.: +86-18354219786

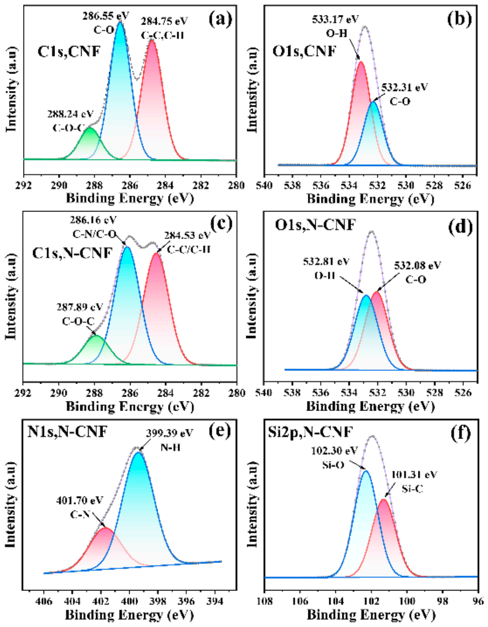

Figure S1. Fitting curves for the regions of C1s (a) and O1s (b) for CNF; C1s (c), O1s (d), N1s (e) and Si2p (f) for N-CNF.

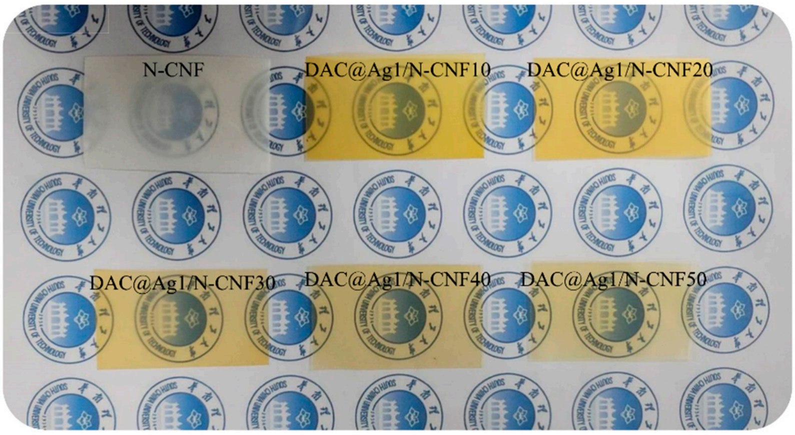

Figure S2. Physical diagram of N-CNF film and DAC@Ag1/N-CNF10~DAC@Ag1/N-CNF50 composite films.

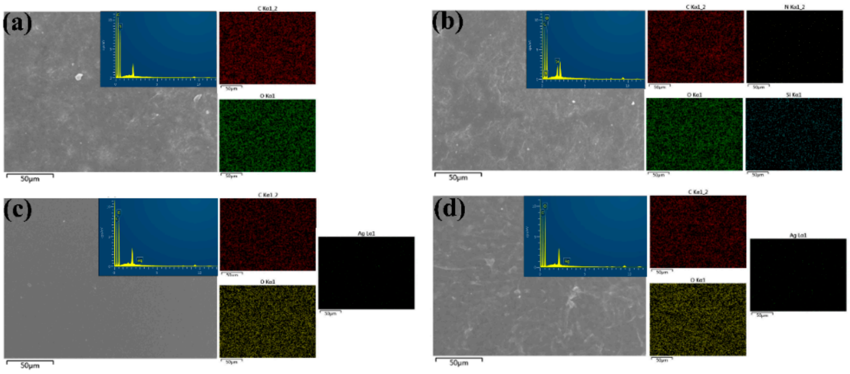

Figure S3. EDS elemental analysis of (a) CNF, (b) N-CNF, (c) DAC@Ag1 and (d) DAC@Ag1/CNF50 films.

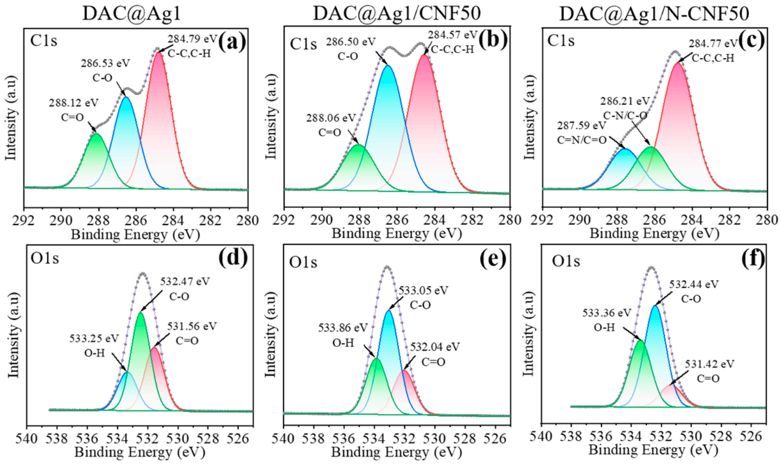

Figure S4. XPS fitted spectra of different films: (a), (b), (c) C1s region, (d), (e), (f) O1s region region for DAC@Ag1, DAC@Ag1/CNF50 and DAC@Ag1/N-CNF50 composite films.

Table S1. Barrier properties of CNF, N-CNF, DAC@Ag1, DAC@Ag1/N-CNF10 to DAC@Ag1/N-CNF50 and DAC@Ag1/CNF50 films.

Samples	WVT（$g/m^2·24\ h$）	OTR（$cm^3/m^2·24\ h·0.1$ MPa）
CNF	186.54±3.54	5.77±0.35
N-CNF	175.48±4.12	4.59±0.30
DAC@Ag1	140.41±4.52	＜0.02
DAC@Ag1/N-CNF10	149.74±3.99	3.63±0.26
DAC@Ag1/N-CNF20	156.55±4.81	3.11±0.37
DAC@Ag1/N-CNF30	162.54±5.75	1.95±0.58
DAC@Ag1/N-CNF40	169.46±3.50	＜0.02
DAC@Ag1/N-CNF50	171.56±6.11	＜0.02
DAC@Ag1/ CNF50	178.53±2.57	3.31±0.49

Table S2. Antibacterial properties of CNF, N-CNF, DAC@Ag1, DAC@Ag1/N-CNF10 to DAC@Ag1/N-CNF50 and DAC@Ag1/CNF50 films.

Samples	inhibition zone diameter（mm）		inhibition rate（%）	
	E. coli	*S. aureus*	*E. coli*	*S. aureus*
CNF	0	0	0	0
N-CNF	0.13±0.08	0.22±0.11	28.43±2.01	29.31±2.59
DAC@Ag1	3.05±0.17	2.76±0.15	90.01±0.58	90.39±0.92
DAC@Ag1/N-CNF10	6.88±0.31	12.15±0.21	99.98±0.02	＞99.99
DAC@Ag1/N-CNF20	7.06±0.23	13.08±0.35	99.99±0.01	＞99.99
DAC@Ag1/N-CNF30	7.20±0.34	13.86±0.26	＞99.99	＞99.99
DAC@Ag1/N-CNF40	7.57±0.19	14.05±0.58	＞99.99	＞99.99
DAC@Ag1/N-CNF50	7.93±0.25	15.91±0.49	＞99.99	＞99.99
DAC@Ag1/ CNF50	1.94±0.28	2.33±0.38	87.26±1.29	88.14±1.33

Table S3. This study compares with other wound healing products in Inhibition Zone diameter.

Samples	*E.coli*(mm)	*S.aureus*(mm)	Ref
licorice extract	-	7	[1]
nonwoven wound dressings coated with MH or MG	> 1	> 1	[2]
PolyacrylonitrileMoringa Extract Nanofibers	15	12	[3]
PGC4	13	14.55	[4]
ssDNA-AgNPs@GO	12.83	8.6	[5]
Salvianic acid A	8.53	11.75	[6]
2.5%PVP-1	-	12	[7]
UVC-LED	-	5.43	[8]
5 wt% CD-loaded electrospun PVA nanofiber	-	10.00	[9]
BAC/KAO	-	11.295	[10]
DAC@Ag1/N-CNF50	7.95	15.95	This work

Table S4. Barrier properties of CNF, N-CNF, DAC@Ag1, DAC@Ag1/N-CNF10 to DAC@Ag1/N-CNF50 and DAC@Ag1/CNF50 films and their statistical data.

Samples	WVT<br>(g/m²·24 h)	ORT<br>(cm³/m²·24 h·0.1 MPa)
CNF	186.54±3.54	5.77±0.35
N-CNF	175.48±4.12	4.59±0.30
DAC@Ag1	140.41±4.52	< 0.02
DAC@Ag1/N-CNF10	149.74±3.99	3.63±0.26
DAC@Ag1/N-CNF20	156.55±4.81	3.11±0.37
DAC@Ag1/N-CNF30	162.54±5.75	1.95±0.58
DAC@Ag1/N-CNF40	169.46±3.50	< 0.02
DAC@Ag1/N-CNF50	171.56±6.11	< 0.02
DAC@Ag1/ CNF50	178.53±2.57	3.31±0.49
F	32.777	123.204
P	< 0.001	< 0.001

Table S5. Water absorption and contact angle of CNF, N-CNF, DAC@Ag1, DAC@Ag1/N-CNF10 to DAC@Ag1/N-CNF50 and DAC@Ag1/CNF50 films and their statistical data.

Sample	Water absorption(%)	Contact angle(°)
CNF	88.63±1.71	70.69±2.27
N-CNF	74.56±2.61	73.87±1.67
DAC@Ag1	3.31±0.10	66.63±0.39
DAC@Ag1/N-CNF10	33.79±0.93	78.28±1.66
DAC@Ag1/N-CNF20	37.88±0.62	84.71±3.61
DAC@Ag1/N-CNF30	38.66±1.12	88.00±2.38
DAC@Ag1/N-CNF40	38.92±1.36	89.20±2.82
DAC@Ag1/N-CNF50	40.85±0.83	89.36±2.47
DAC@Ag1/ CNF50	51.85±1.36	69.36±3.57
F	980.119	39.936
P	< 0.001	< 0.001

Table S6. Inhibition Zone diameter of CNF, N-CNF, DAC@Ag1, DAC@Ag1/N-CNF10 to DAC@Ag1/N-CNF50 and DAC@Ag1/CNF50 films and their statistical data.

Sample	E.coli(mm)	S.aureus(mm)
CNF	0	0
N-CNF	0.13±0.08	0.22±0.11
DAC@Ag1	3.05±0.17	2.75±0.15
DAC@Ag1/N-CNF10	6.85±0.31	12.05±0.21
DAC@Ag1/N-CNF20	7.05±0.23	13.00±0.35
DAC@Ag1/N-CNF30	7.20±0.34	13.85±0.26
DAC@Ag1/N-CNF40	7.55±0.19	14.05±0.58
DAC@Ag1/N-CNF50	7.95±0.26	15.95±0.49
DAC@Ag1/ CNF50	1.94±0.28	2.33±0.38
F	618.687	1224.685
P	< 0.001	< 0.001

Let α=0.05, the P-values of the samples in barrier properties, water absorption, contact angle and Inhibition Zone diameter as shown in the Table S4 to Table S6, are all below 0.001, indicating significant statistical differences in the results obtained from the aforementioned experimental tests. That is, the samples from various groups exhibit notable distinctions in barrier properties, water absorption, contact angle, and inhibition zone diameter.

References:

1. Hasan, M.M.; Shahid, M.A. Pva, licorice, and collagen (plc) based hybrid bio-nano scaffold for wound healing application. *Journal of Biomaterials Science-Polymer Edition* **2023**, *34*, 1217-1236.
2. Bulman, S.; Tronci, G.; Goswami, P.; Carr, C.; Russell, S. Antibacterial properties of nonwoven wound dressings coated with manuka honey or methylglyoxal. *Materials* **2017**, *10*, 954.
3. Fayemi, O.E.; Ekennia, A.C.; Katata-Seru, L.; Ebokaiwe, A.P.; Ijomone, O.M.; Onwudiwe, D.C.; Ebenso, E.E. Antimicrobial and wound healing properties of polyacrylonitrile-moringa extract nanofibers. *Acs Omega* **2018**, *3*, 4791-4797.
4. Rashid, N.; Khalid, S.H.; Ullah Khan, I.; Chauhdary, Z.; Mahmood, H.; Saleem, A.; Umair, M.; Asghar, S. Curcumin-loaded bioactive polymer composite film of pva/gelatin/tannic acid downregulates the pro-inflammatory cytokines to expedite healing of full-thickness wounds. *Acs Omega* **2023**, *8*, 7575-7586.
5. Tong, C.; Zou, W.; Ning, W.; Fan, J.; Li, L.; Liu, B.; Liu, X. Synthesis of dna-guided silver nanoparticles on a graphene oxide surface: enhancing the antibacterial effect and the wound healing activity. *Rsc Advances* **2018**, *8*, 28238-28248.
6. Huang, T.; Lin, M.; Lin, M.; Lou, C.; Chen, Y.; Lin, J. Antibacterial wound dressings made of differently concentrated salvia miltiorrhiza bunge via electrospinning. *Journal of Polymer Research* **2023**, *30*.
7. Nagle, A.; Kopel, J.; Reed, J.; Jacobo, U.; Tran, P.; Mitchell, K.; Reid, T.W. An in vitro study of betadine's ability to eliminate live bacteria on the eye: should it be used for protection against endophthalmitis? *Antibiotics* **2022**, *11*, 1549.
8. Sheikh, J.; Swee, T.T.; Saidin, S.; Yahya, A.B.; Malik, S.A.; Yin, J.; Thye, M. Bacterial disinfection and cell assessment post ultraviolet-c led exposure for wound treatment. *Medical & Biological Engineering & Computing* **2021**, *59*, 1055-1063.
9. Alabdali, A.Y.M.; Khalid, R.; Kzar, M.; Ezzat, M.O.; Huei, G.M.; Hsia, T.W.; Mogana, R.; Rahman, H.; Razik, B.M.A.; Issac, P.K.; et al. Design, synthesis, in silico and antibacterial evaluation of curcumin derivatives loaded nanofiber as potential wound healing agents. *Journal of King Saud University - Science* **2022**, *34*, 102205.
10. Zhang, X.; Li, S.; Deng, Y.; Zuo, Z.; Sun, Z.; Li, C.; Zheng, S. Benzalkonium chloride modified kaolinite with rapid bactericidal activity for the development of antibacterial films. *Materials Today Communications* **2023**, *35*, 106152.
